# Advances in culture, expansion and mechanistic studies of corneal endothelial cells: a systematic review

**DOI:** 10.1186/s12929-018-0492-7

**Published:** 2019-01-04

**Authors:** Shuangling Chen, Qin Zhu, Hong Sun, Yuan Zhang, Sean Tighe, Li Xu, Yingting Zhu

**Affiliations:** 1 Tissue Tech, Inc., 7235 Corporate Center Drive, Suite B, Miami, Florida 33126 USA; 2 Department of Ophthalmology, Fourth Affiliated Hospital of Kunming Medical University (the Second People’s Hospital of Yunnan Province), Key Laboratory of Yunnan Province for the Prevention and Treatment of Ophthalmology, Provincial Innovation Team for Cataract and Ocular Fundus Disease, The Second People’s Hospital of Yunnan Province, Expert Workstation of Yao Ke, Yunnan Eye Institute, Kunming, 650021 China; 3 Department of Ophthalmology, the First Affiliated Hospital of Nanjing Medical University, Nanjing, 210029 China; 4 The Department of Ophthalmology, The Affiliated Hospital of Inner Mongolia Medical University, Tongdao North Rd, Hohhot, Inner Mongolia China

**Keywords:** Cornea, Endothelium, Expansion, Mechanism

## Abstract

Human corneal endothelial cells are notorious for their restricted proliferative ability in vivo and in vitro. Hence, injury or dysfunction of these cells may easily result in blindness. Currently, the only treatment is to transplant a donor cornea that contains a healthy corneal endothelium. However there is a severe global shortage of donor corneas and there remains an unmet clinical need to engineer human corneal grafts with healthy corneal endothelium. In this review, we present current advances in the culture, expansion, and molecular understandings of corneal endothelial cells in vitro in order to help establish methods of engineering human corneal endothelial grafts.

## Background

The front part of the eye (i.e. cornea) is one of the most important human organs. In particular, the corneal endothelial cells are a vital component that regulate nutrition and hydration to maintain clear vision. Unfortunately, these cells are notorious for their limited proliferative ability. Consequently, loss in cell number or function caused by aging, injury or surgery (termed corneal endothelial dysfunction) can lead to corneal blindness. Treatment of this dysfunction traditionally requires a corneal transplant utilizing cadaver donor tissue however there is an increasing global shortage of donor corneal tissues. Therefore, it is paramount to find a replacement solution. In order to resolve this urgent problem, many scientists have researched alternatives instead of using cadaver tissue. One of the emerging methods is to use fetal or adult corneal stem cells/progenitors (which inherently have proliferative capabilities) to engineer appropriate corneal tissue grafts for surgical therapies. Another method is to reprogram adult human corneal cells to their progenitor status and then creating surgical grafts. Overall, these developments in human corneal stem cell therapies may lead to new discoveries in human corneal regenerative medicine and provide mechanistic understanding for other fields of medicine.

### Substrates required for effective expansion of corneal endothelial cells (CEC)

Collagen IV is basement membrane-specific collagen which was found in the endothelial and epithelial layers [62], suggesting collagen IV is important for endothelial structure and functions. Since the first discovery of collagen IV by Kefalides in 1966, collagen IV has been investigated extensively by many research laboratories around the world. So far, six evolutionary related mammalian genes encoding six different polypeptide chains of collagen IV α-chain polypeptides (α1–α6) have been identified and characterized [22]. The NC1 domain (a long triple-helical collagenous domain and a globular non-collagenous C4 domain at the N and C terminus) is considered important for the assembly of the type IV collagen trimeric structure. The assembly of the trimer begins when three NC1 domains initiate a molecular interaction between three α-chains. Protomer trimerization proceeds like a zipper from the carboxy-terminal end, resulting in a fully assembled protomer and dimer formation of type IV collagen.

Collagen IV is very important to maintain the normal phenotype of CEC and prevent endothelial-mesenchymal transition (EMT). For example, bovine corneal endothelial cells lose their phenotype on glass or tissue culture polystyrene without Collagen IV coating. Additionally bovine corneal endothelial phenotype lose their phenotype when cultured on fibronectin and collagen I coating. In contrast, with collagen IV coating, the endothelial cells exhibit polygonal morphology, ZO-1 at the cell border, and F-actin located cortically [48], suggesting collagen IV plays a critical role in maintaining the phenotype of endothelial cells [37]. In addition, collagen IV may facilitate attachment and promote expansion of CEC [3, 80]. Consistent with this view, normal endothelial cells produce collagen IV as the major type of collagen while fibroblastic corneal endothelial cells produce collagen I as the major type of collagen [23]. Although collagen IV exerts unique functions for corneal endothelium as mentioned above, the mechanism remains unclear how collagen IV uniquely affects the behavior of human CEC aggregates (not single cells) [35, 36, 75–78].

### Media and growth factors for expansion of human corneal endothelial cells (HCEC)

A number of cell culture media have been used for growth of HCECs, including DMEM, DMEM and F12, Ham’s F12 and M199, and Opti-MEM-I [44]. HCECs cultured in DMEM or DMEM and F12 could not be passaged more than once or twice [51]. On the contrary, HCECs expanded in Ham’s F12 and M199 or Opti-MEM-I could be passaged up to 3 times (reviewed in [44]) with HCEC markers, for example, Na + K+/ATPase and ZO-1 (reviewed in [44]). However, the cultured HCEC were not hexagonal in shape suggesting these medias are not optimal. In other reports, MEM, MESCM, and OptiMEM-I with 8% FBS, 20 ng/mL NGF, 5 ng/mL EGF have been found to be effective [19, 74, 79].

Growth Factors such as bFGF [7, 10–12, 57, 69], EGF [7, 33, 57, 58, 69, 75, 77], LIF [35, 79], NGF [7], may stimulate expansion of HCEC. We have reported that LIF delays contact-inhibition and is significantly more effective with bFGF without serum to promote HCEC growth without change of phenotype [35, 79].

### Corneal differentiation from ESCs

Many cell types can be differentiated into HCEC [49]. CEC-like cells can be differentiated from hESCs [31]. The differentiation of hESCs to HCEC occurs via the periocular mesenchymal precursor (POMP) by a two-step inducement [7, 49]. The cells obtained from this method were characteristics of HCEC [71]. Cord blood-derived MSCs might also be differentiated into HCEC-like cells [20].

### Traditional expansion of CEC

Traditionally, disruption of the contact inhibition has been adopted to unlock the mitotic block of CEC through the use of EDTA ± trypsin to break cell-cell junctions [50, 59]. In the presence of basic fibroblast growth factor (bFGF) [24, 26, 30, 32] or epithelial growth factor (EGF) [53], this conventional method has been used by several laboratories to promote human CEC proliferation as a strategy to engineer CEC grafts for tissue transplantation [6, 10, 13, 17, 18, 28, 35, 41, 43, 60, 68]. However, according to many studies including those conducted by the laboratory of Dr. Eundak Kay [26], this conventional strategy causes pathologic endothelial mesenchymal transition (EMT) [30, 32].

### Mechanism of EMT

The key events of EMT in disruption of cell-cell junctions are: 1. loss of apical–basal polarity and acquisition of a front–rear polarity; 2. reorganization of the cytoskeletal architecture and changes in cell shape; 3. downregulation of an epithelial gene expression signature, activation of mesenchymal phenotype genes, increased cell protrusions and motility; and, 4. in many cases, an ability to degrade extracellular matrix proteins to enable invasive behavior [29, 39].

During the destabilization of adherent junctions, cell junctional proteins are cleaved at the plasma membrane and subsequently degraded or relocated [67]. Epithelial cadherin (E-cadherin) is cleaved at the plasma membrane and subsequently degraded. Consequently, β-catenin can no longer interact with E-cadherin and it is either degraded or protected from degradation (for example, in response to WNT signaling), so that it can act in transcription [45]. p120 catenin (also known as catenin δ1) also accumulates in the nucleus and participates in transcription following a decrease in E-cadherin levels [27]. In addition, the downregulation of E-cadherin is balanced by the increased expression of N-cadherin, which results in a ‘cadherin switch’ that alters cell adhesion [64, 67]. Through this switch, the transitioning cells lose their association with epithelial cells and acquire an affinity for mesenchymal cells through homotypic N-cadherin interactions, facilitating cell migration and invasion.

Cells that undergo EMT reorganize their cortical actin cytoskeleton into one that enables dynamic cell elongation and directional motility [61, 67]. New actin-rich membrane projections facilitate cell movement and act as sensory extension of the cytoskeleton. These projections include sheet-like membrane protrusions called lamellipodia and spike-like extensions called filopodia at the edge of lamellipodia [54]. EMT is characterized by increased cell contractility and actin stress fiber formation. These dynamic changes in actin organization are probably mediated by regulatory proteins such as moesin [14], but the molecular mechanisms controlling F-actin dynamics during EMT remain to be elucidated. Keratin and vimentin filaments regulate the trafficking of organelles and membrane-associated proteins, but they show differences in the proteins that they target to the membrane. Changes in intermediate filament composition also enable cell motility, possibly because of the interaction of vimentin with motor proteins [40]. However, how change of cytoskeleton is linked to change of cell-cell junctions and how the change of cytoskeleton and cell-cell junctions is linked to EMT remain unclear.

The change of cytoskeleton, actin dynamics and control of actin rearrangement during EMT are at least partially regulated by Rho GTPases. Among these, RhoA promotes actin stress fiber formation, whereas RAC1 and CDC42 mainly promote the formation of lamellipodia and filopodia. Rho GTPases also regulate the formation of cell–cell junction complexes and the stability of adherent junctions. After dissolution of cell-cell junctions, cytoplasmic p120 dissociates from the cell membrane and represses RhoA activity but activates RAC and CDC42 to induce the formation of membrane protrusions and cell motility [2]. In addition, RAC1 promotes microtubule polymerization and is activated by itself at the leading edge of cells, which sets up a positive feedback loop that participates in the reorganization of the microtubule cytoskeleton [66]. The reorganization of the cytoskeletal architecture and polarity complexes, which result in cell shape changes, cell elongation, membrane protrusions and front–rear polarity, are essential in EMT and enable directional migration. These results suggest balance of RhoA and Rac1 are major regulators of EMT. However, we do not know exactly how RhoA and Rac1 regulate EMT.

### Novel approach for expansion of CEC without EMT

A novel strategy of effective expansion of human CEC monolayer with normal cell phenotype has been established by collagenase digestion (without disruption of cell-cell junctions) and p120-Kaiso knockdown (effective expansion). Because collagenase digestion selectively removes interstitial but not the basement membrane collagens, this method does not disrupt intercellular junctions nor interaction with the basement membrane. Indeed, collagenase digestion results in compact aggregates of human CEC, which retain intercellular junctions mediated by ZO-1 and connexin-43, and maintain their adhesion to such basement membrane components as collagen IV, laminin 5, and perlecan [33]. Using p120-Kaiso siRNA knockdown, human CEC monolayers have been successfully expanded in supplemental hormonal epithelial medium (SHEM) to an average size of 5.0 ± 0.4 mm in diameter from Descemet membrane stripped from 1/8 of the corneoscleral rim [75]. Knockdown by p120 siRNA selectively activates p120/Kaiso signaling as evidenced by nuclear translocation of p120 to release nuclear Kaiso (a repressor) and to induce colocalization of nuclear bromodeoxyuridine (BrdU) labeling [5, 75]. The knockdown regimen of p120 siRNA regarding dosing, frequency, and starting time has been further optimized and this optimized p120 siRNA knockdown is synergized by addition of Kaiso siRNA knockdown to further expand the size of human CEC monolayers to 11.0 ± 0.6 mm in diameter [76].

To confirm the phenotype of the generated HCEC, we have performed immunostaining for HCEC markers. The specific corneal endothelial markers include cytoplasmic γ-tubulin and p75NTR and junctional α-catenin, β-catenin, F-actin, N-cadherin, Na-K-ATPase, and p120 (all are markers of HCEC), without expression of LEF1 and S100A4 (both are markers of EMT) [75, 76, 78]. Such expression of HCEC markers was consistent with the in vivo expression pattern previously reported by us [75, 76, 78].

### Mechanism of reprogramming CEC without EMT

Previously, it is reported that mitotic block of human CEC due to contact inhibition is established after three weeks of culturing in an EGF- and serum-containing medium termed SHEM [77]. However, a switch from SHEM to a serum-free LIF-containing medium termed MESCM delays contact inhibition by 2 weeks [35]. The delay in contact inhibition may permit human CEC to be responsive to reprogramming into NC progenitors by p120-Kaiso signaling, resulting in further expansion of human CEC monolayers to a transplantable size [78].

Subsequent investigation suggests that the expansion of human CEC in SHEM is closely correlated with the activation of RhoA signaling to promote BrdU labeling, which requires nuclear translocation of nuclear factor κB (pNFκB, p65, S276) [76]. Inhibition of RhoA by CT-04, ROCK by Y27632, bone morphological protein (BMP) by Noggin, TAK1 by 5Z-7-oxozeaenol, or NFκB by CAY10512 abolished nuclear translocation of pNFκB, which is required for nuclear translocation of p120 and BrdU labeling [76]. The nuclear translocation of pNFκB is mediated by BMPRI-TAK1-XIAP complex as a result of activation of the non-canonical BMP signaling [16, 18], evidenced by upregulation of BMP2, BMP4, BMPR1A, and BMPR1B transcripts but not nuclear location of pSmad1/5/8, and the lack of activation of inhibitor of differentiation 1–4 (ID1–4), which are known targets of nuclear Smads triggered by the canonical BMP signaling [76]. Interestingly, activation of canonical BMP signaling by p120-Kaiso siRNAs requires a switch of medium from EGF-containing serum-containing SHEM to leukemia Inhibitory Factor (LIF)-containing serum free MESCM [76]. As proposed, p120 acts, at least in part, through regulation of Rho GTPases and its downstream target ROCK1/2 [1].

**Fig. 1:**
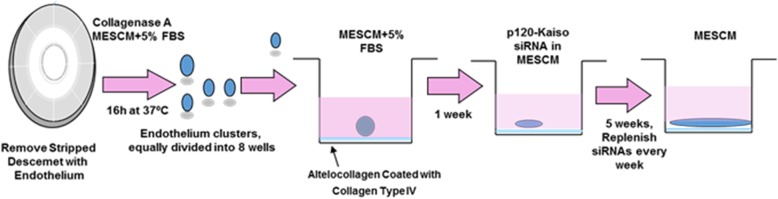
Novel approach for expansion of HCEC without EMT

RhoA-GTP can be activated by p120 siRNA and further by p120-Kaiso siRNAs [76]. This novel approach is summarized in Fig. 1. Activation of p120/Kaiso-RhoA-ROCK pathway may promote nuclear translocation of p120 to relieve the repressor activity of Kaiso, a member of BTB/POZ-ZF transcription factor family without disruption of cell-cell junctions, without activation of canonical Wnt signaling and thus without EMT [25, 55, 70, 75, 76], (also reviewed in [8]). Because activation of RhoA-ROCK signaling is clearly linked to inhibition of canonical Wnt signaling, we believe that inhibition of RhoA-ROCK signaling may relieve inhibition of canonical Wnt signaling and as a result, cause EMT.

**Fig. 2:**
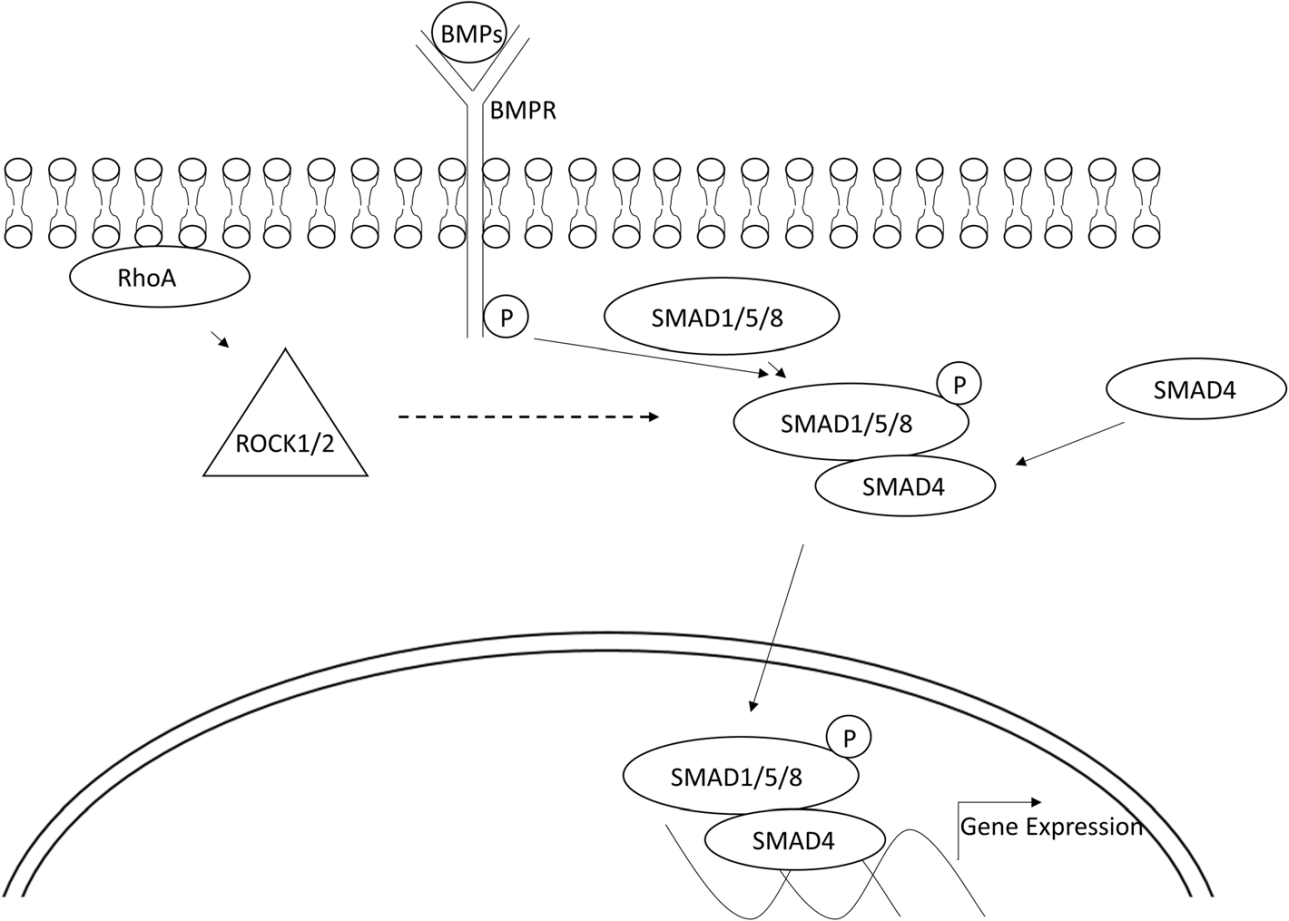
RhoA-ROCK-canonical BMP signaling in reprogramming of HCEC

It remains unclear how p120-Kaiso siRNAs leads to activation of canonical BMP signaling. As reported [75, 76], weekly knockdown with p120-Kaiso siRNAs activates RhoA-ROCK signaling. RhoA may regulate the actin cytoskeleton and the expression of genes associated with cell proliferation. As suggested, RhoA may upregulate cJUN expression through ROCK (independently from the ability of ROCK to promote actin polymerization), which either indirectly via activation [38] of JNK or directly via phophorylation of cJUN and ATF2, which then bind with the AP1 site of cJUN promoter in NIH 3 T3 and HEK293T cells to promote cJUN expression [38]. DNA sequence analysis has revealed 4 AP-1 sites and 12 AP-1 sites in the BMP4 and BMP6 promoter, respectively (ENCODE data at UCSC). Because overexpression of cJUN promotes BMP4 transcription in Xenopus embryos, and because the AP-1 site serves as additional activatory component for the auto-regulation of BMP4 [9, 21, 56], we believe that JNK-cJUN signaling serves as a “missing” link for p120-RhoA-ROCK signaling to activate canonical BMP signaling that is required to promote the reprogramming of human CEC to progenitors. The RhoA-ROCK-canonical BMP signaling is summarized in Fig. 2.

p16^INK4a^ belongs to the family of cyclin-dependent kinase inhibitors (CKIs) [4]. Nuclear translocation of phosphorylated p16^INK4a^ is one hallmark for CEC senescence, an irreversible arrest during the G1 transition of the cell cycle [15, 47, 52]. This a notion consistent with what has been previously reported [42] and senescence has been recognized as a barrier for reprogramming into iPSC [4, 15, 34]. Therefore, it is important to study whether nuclear exclusion of p16^INK4a^ is critical for reprogramming adult human CEC into neural crest progenitors, and if so, how nuclear exclusion of p16^INK4a^ is achieved. JAK2 signaling may serve as a “missing” link for p120-RhoA-ROCK signaling to activate STAT3 Tyr 705 signaling which is required to promote reprogramming of human CEC to progenitors via activation of Bmi1-mediated inhibition (Bmi1 is an inhibitor of p16) of p16-mediated senescence.

### Lineage-tracing technology

Zhao et al. have reported corneal endothelial cells could be induced from human iPSCs [73], resulting in differentiation of iPSCs into corneal stem cells by expressing corneal lineage transcription factors, LHX2, PAX6, and VSX2 and subsequent expression of corneal neural crest stem cells and corneal endothelial cells (reviewed in [63]). Therefore, it is assumed that a subset of neural crest cells are corneal specific and can be corneal endothelial cells. Zhao et al. also reported generation of HCEC from iPSCs obtained from human foreskin fibroblasts [73]. Another study obtained iPSCs from human dermal fibroblasts [65], suggesting that we may obtain corneal endothelial cells from human iPSCs. We have reported that HCEC can be induced from human trabecular meshwork cells [72]. However, it remains unclear whether the stem cell-CEC resemble mature HCEC? To our knowledge, it is best to obtain HCEC from a subset of HCEC progenitors [76, 78], not from other origins [5], because those cells are more phenotypic than other HCEC induced from iPSCs or other resources.

### Prospect of engineering of human CEC grafts for clinical applications

In the clinical application of cultured HCEC, many issues require consideration. For example, unwanted issues associated with application of cultured HCEC are usually linked to the microenvironment in which cultured HCEC engraft, causing unfavorable differentiation of transplanted cultured cells. This may be caused by many factors including unfavorable substrates, isolation method (substrates other than collagen IV, isolation method EDTA and trypsin), media for cell culture (culture media other than MESCM) as we have previously tested and reported (reviewed in [80]), or tumor growth (reviewed in [46]). In addition, applications of cultured HCEC may require immune-suppression for prevention of rejection of cultured HCEC grafts as we have experienced in a mini pig model. Furthermore, anatomical non-limbus HCEC, for example, human central CEC are extremely difficult to expand, while human peripheral CEC are expandable. However, it remains unclear whether HCEC expanded from peripheral areas are functional as those in the central area.

Pre-clinical animal models are required for the examination of the safety and efficacy of engineered corneal endothelial grafts. The current challenges for engineering human CEC are the difficulties for effective isolation and expansion of CEC without EMT. Still there are no cell-based therapies to cure human corneal endothelial dysfunction up to now. Therefore, it is important to strive for resolving the engineering issues of human CEC grafts in the near future.

## Conclusions

This review has summarized the latest progress in substrate selection, in vitro expansion, and tissue engineering of human corneal endothelial grafts. Based on recent progress, the future is bright and this novel technique may generate a new treatment of CEC dysfunction and human blindness.

## Abbreviations

bFGF: Basic fibroblast growth factor
BMP: Bone morphological protein
BrdU: Bromodeoxyuridine
C4: Domain at the N and C terminus
CEC: Corneal endothelial cells
CKI: Cyclin-dependent kinase inhibitor
E-cadherin: Epithelial cadherin
EGF: Epithelial grow factor
EMT: Endothelial-mesenchymal transition
GAP: GTPase-activating protein
GEF: Guanine nucleotide exchange factor
HCEC: Human corneal endothelial cells
ID: Inhibitor of differentiation
JAK: Janus kinase/signal transducer
JNK: Jun N-terminal kinase
LIF: Leukemia Inhibitory Factor
MAPK: Mitogen-activated protein kinase
NC1 domain: A long triple-helical collagenous domain and a globular non-collagenous
N-cadherin: Neural cadherin
NFκB: Nuclear factor κB
p16^INK4a^: Cyclin-dependent kinase 4 inhibitor A
SHEM: Supplemental hormonal epithelial medium
STAT: Activators of transcription
ZO-1: Tight junction protein 1
